# Multiple Myeloma in a 45-Year-Old Woman with Screening-Detected Anemia and Unintentional Weight Loss: A Case Report

**DOI:** 10.3390/reports9040336

**Published:** 2026-10-06

**Authors:** Kosuke Mori, Hidehiro Someko, Miyuki Ando

**Affiliations:** 1 Department of Internal Medicine, Nagoya Tokushukai General Hospital, 2-52 Kozoji-cho-kita, Kasugai 487-0016, Aichi, Japan; 2 SRWS-PSG, Osaka 530-0001, Osaka, Japan; 3 Department of Cardiology, Nagoya Tokushukai General Hospital, 2-52 Kozoji-cho-kita, Kasugai 487-0016, Aichi, Japan

**Keywords:** multiple myeloma, anemia, health screening, unintentional weight loss, case report

## Abstract

**Background and Clinical Significance**: Anemia is commonly detected during health screening and may initially appear to be an isolated hematologic abnormality. Careful history-taking is important because associated symptoms may provide clues to serious underlying disease. We report a case of multiple myeloma in which unintentional weight loss was the key clinical clue during evaluation of screening-detected anemia. **Case Presentation**: A 45-year-old woman was referred for evaluation of anemia detected during routine health screening. Initial laboratory testing showed normocytic anemia without evidence of iron deficiency, vitamin B12 deficiency, or folate deficiency. Routine history-taking for anemia revealed unintentional weight loss of 3.5 kg (4.6% of her body weight) over three months, prompting evaluation for malignancy. Non-contrast computed tomography demonstrated an osteolytic lesion in the manubrium. Serum protein electrophoresis showed an M-peak, and immunofixation detected an IgA-kappa monoclonal protein and urinary kappa-type Bence Jones protein. Serum free light-chain testing revealed a markedly elevated kappa/lambda ratio. At the referral hematology department, bone marrow aspiration showed 77.8% plasma cells, and biopsy and clot sections showed extensive infiltration by CD138-positive plasma cells, consistent with plasma cell myeloma. She subsequently received chemotherapy at the referral hospital, achieved remission, and has returned to work. **Conclusions**: Unintentional weight loss can serve as an important red flag when evaluating non-iron-deficiency anemia detected through health screening. Actively eliciting weight change may facilitate prompt diagnosis of an otherwise unsuspected hematologic malignancy.

## 1. Introduction and Clinical Significance

In Japan, health screening is widely implemented through periodic workplace health examinations, various health checkups conducted by local governments and insurers, and comprehensive medical checkups, with the aim of disease prevention and early detection [1].

Anemia is a common finding at health screening. In a study of 3385 women undergoing Ningen Dock (comprehensive health checkup) examinations, low hemoglobin requiring further evaluation, defined as hemoglobin ≤ 11.0 g/dL, was observed in 9.7% overall. By age group, the prevalence was 8.7% among women in their 30s and 16.6% among those in their 40s, indicating that anemia is not uncommon among premenopausal women [2]. In this clinical context, anemia in premenopausal women is often initially attributed to iron deficiency, mainly because of physiological iron loss associated with menstruation [3,4].

Normocytic normochromic anemia has a broad differential diagnosis, including renal disease, chronic inflammatory disorders, hemolysis, and bone marrow disorders. When anemia is accompanied by unintentional weight loss, an underlying malignancy should also be considered [5,6]. In plasma cell disorders such as multiple myeloma, anemia may coexist with monoclonal protein abnormalities and osteolytic bone lesions; this combination should prompt targeted evaluation for a plasma cell neoplasm [7].

We report the case of a 45-year-old woman who presented for evaluation of anemia detected during health screening. Although her anemia was normocytic, further laboratory evaluation did not support iron deficiency or vitamin B12 or folate deficiency. Detailed history-taking revealed an unintentional weight loss of approximately 5% over three months. This prompted further evaluation for malignancy and ultimately led to the diagnosis of multiple myeloma.

## 2. Case Presentation

A 45-year-old woman presented to our hospital for evaluation of anemia detected during health screening, with a hemoglobin level of 7.5 g/dL and a mean corpuscular volume (MCV) of 92.1 fL. As part of our routine history-taking for anemia, we asked about body weight changes and found that she had experienced unintentional weight loss over the preceding three months, with her weight decreasing from 75.4 kg to 71.9 kg (a loss of 3.5 kg, or approximately 4.6%). She had no history of trauma and denied overt bleeding or gastrointestinal symptoms. Her medical history was notable only for migraine, for which she used loxoprofen as needed. She had no history of smoking or alcohol consumption and no relevant allergies.

On physical examination, mild pallor of the palpebral conjunctiva was noted. There was no jaundice or cervical lymphadenopathy. Breath sounds were clear, and no heart murmur was audible. The abdomen was flat and soft without tenderness, and bowel sounds were normal. There was no joint swelling or erythema in the extremities, and no lower-leg edema or skin rash was observed. Her height was 168 cm, weight was 71.9 kg, and body mass index was 25.4 kg/m^2^.

Blood tests showed normocytic anemia, with hemoglobin 7.5 g/dL and MCV 92.1 fL; the reticulocyte count was 38.6 × 10^3^/μL. Other blood cell lineages were preserved, with a white blood cell count of 4.0 × 10^3^/μL and platelet count of 234 × 10^3^/μL. Serum chemistry showed calcium 9.2 mg/dL, total protein 6.8 g/dL, albumin 4.2 g/dL, lactate dehydrogenase 192 U/L, creatinine 0.99 mg/dL (estimated glomerular filtration rate [eGFR], 49 mL/min/1.73 m^2^), and soluble interleukin-2 receptor 347 U/mL. Iron studies showed serum iron 101 μg/dL, total iron-binding capacity 294 μg/dL, and ferritin 180.6 ng/mL. Vitamin B12 and folate levels were 506 pg/mL and 5.5 ng/mL, respectively. These findings argued against iron, vitamin B12, and folate deficiency. The reticulocyte count was inappropriately low for the degree of anemia, indicating hypoproliferative anemia. The erythropoietin level was 83.7 mIU/mL, which was above the reference range and did not suggest erythropoietin deficiency as the main cause of anemia despite the mildly reduced eGFR. Dipstick urinalysis was unremarkable.

**Figure 1:**
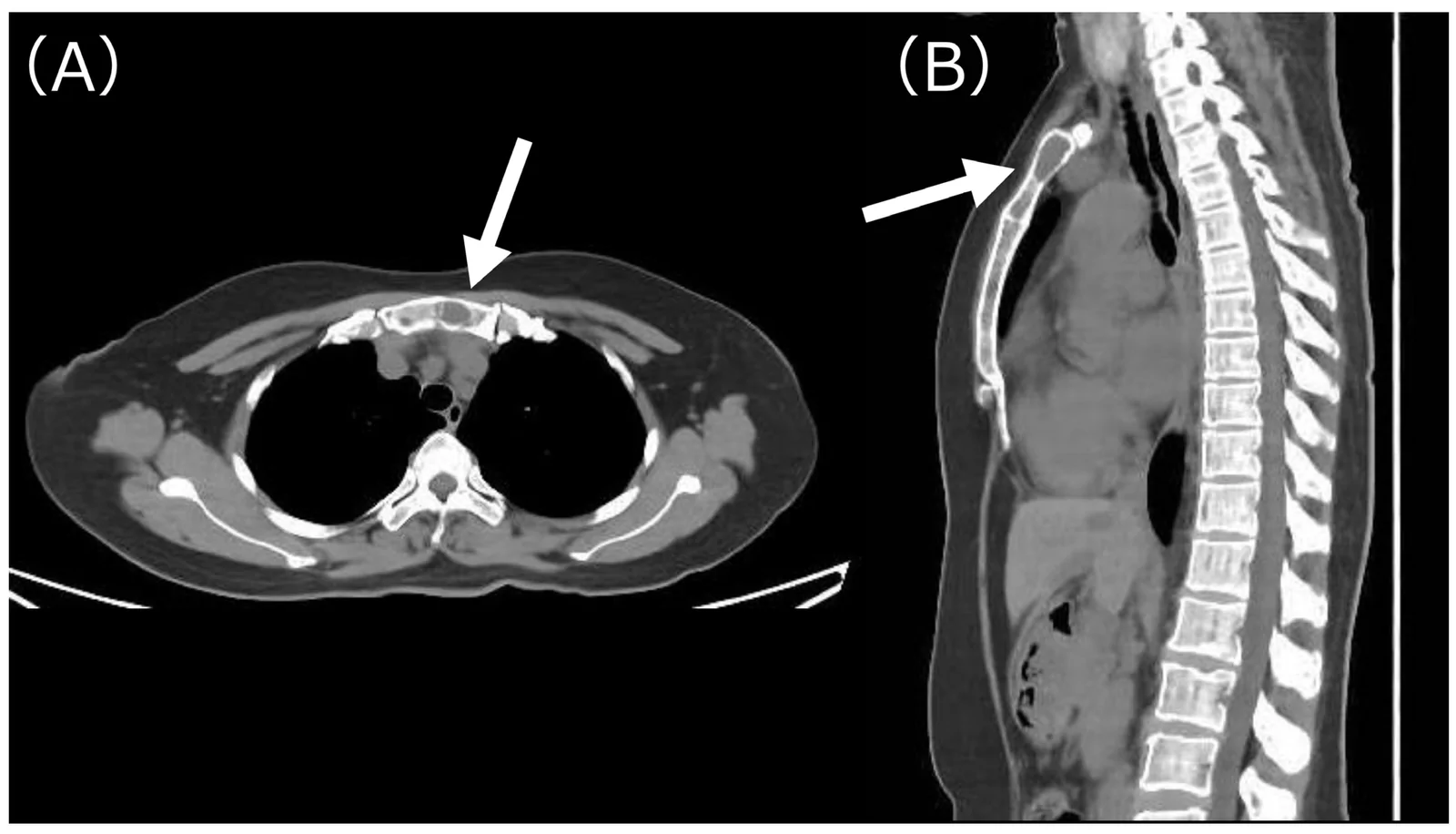
Non-contrast computed tomography showing an osteolytic lesion in the manubrium. (**A**) Axial image. (**B**) Sagittal image. Arrows indicate the lesion.

Because the patient had normocytic normochromic anemia accompanied by unintentional weight loss, malignancy was considered, and non-contrast computed tomography of the chest and abdomen was performed. This revealed an osteolytic lesion in the manubrium suggestive of a bone tumor (Figure 1). Considering the patient’s age and sex, bone metastasis from breast cancer was included in the differential diagnosis; however, breast ultrasonography and mammography showed no apparent abnormalities. Malignant lymphoma was also considered, but lactate dehydrogenase and soluble interleukin-2 receptor levels were not elevated, and imaging did not show systemic lymphadenopathy. The combination of otherwise unexplained normocytic anemia and an osteolytic bone lesion raised suspicion for a plasma cell neoplasm. Therefore, multiple myeloma/plasmacytoma and a primary chest wall or bone tumor were prioritized in the differential diagnosis, and targeted testing for a monoclonal plasma cell disorder was performed.

**Figure 2:**
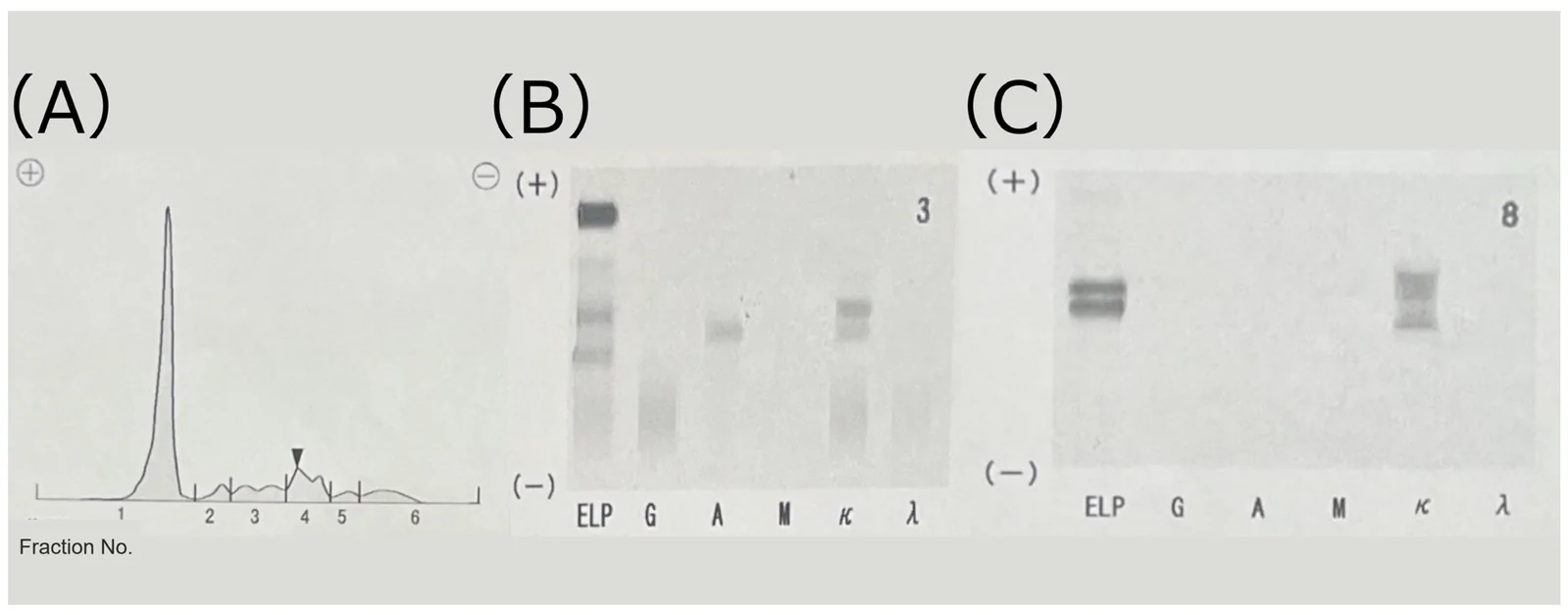
Serum protein electrophoresis and immunofixation demonstrating monoclonal gammopathy. (**A**) Serum protein electrophoresis showing a discrete M-peak outside the conventional gamma region (arrowhead). (**B**) Serum immunofixation demonstrating an IgA-kappa monoclonal protein. (**C**) Urine immunofixation demonstrating kappa-type Bence Jones protein.

Serum protein electrophoresis demonstrated a discrete M-peak outside the conventional gamma region (Figure 2A), a migration pattern commonly observed for IgA monoclonal proteins. Serum immunofixation identified an IgA-kappa monoclonal protein, and urine immunofixation demonstrated kappa-type Bence Jones protein (Figure 2B,C). Serum free light-chain analysis showed kappa 26,775.3 mg/L, lambda 5.4 mg/L, and a kappa/lambda ratio of 4658.39. Because our hospital does not have a hematology department, the patient was referred to the hematology department of another hospital. Bone marrow aspiration showed plasma cells accounting for 77.8% of nucleated cells on morphological differential count, and flow cytometry showed that CD138-positive cells accounted for 67.7% of the analyzed cells. Bone marrow biopsy and clot sections showed that the marrow was almost entirely replaced by CD138-positive plasma cells, with little identifiable normal hematopoietic tissue; the Ki-67 labeling index was 15%, and the pathological diagnosis was plasma cell myeloma. Clonality of the marrow plasma cells was supported by the IgA-kappa monoclonal protein, kappa-type Bence Jones proteinuria, and a serum kappa/lambda ratio of 4658.39, in the setting of near-complete marrow replacement by plasma cells. Based on these findings, a diagnosis of IgA-kappa multiple myeloma was established. She subsequently received chemotherapy at the referral hospital, achieved remission, and has returned to work.

**Figure 3:**
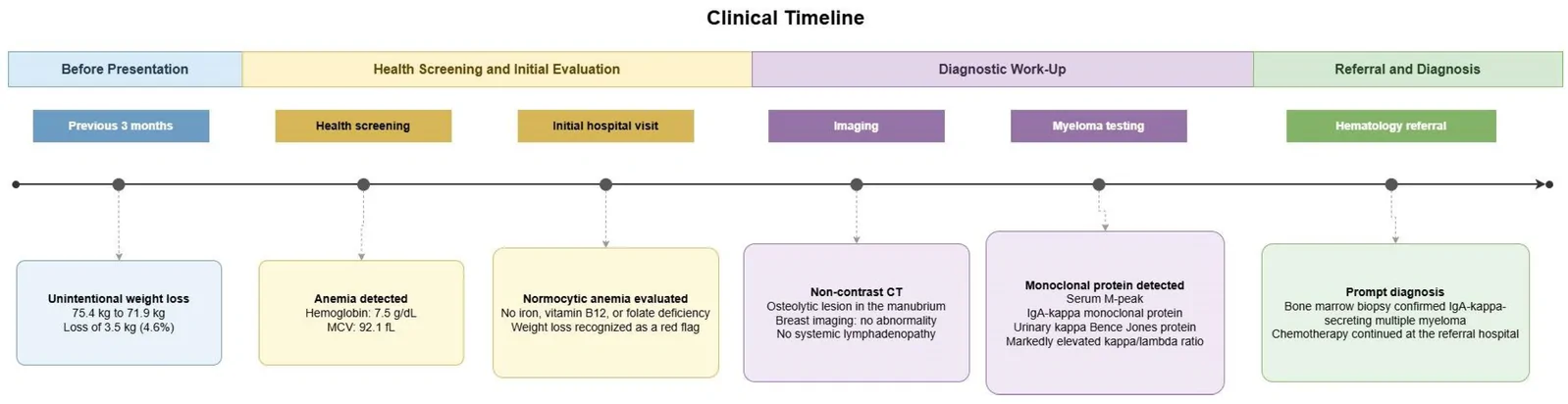
Clinical timeline from unintentional weight loss through health screening, diagnostic work-up, hematology referral, and diagnosis of multiple myeloma. Abbreviations: CT, computed tomography; MCV, mean corpuscular volume.

The clinical course is summarized in Figure 3.

### Patient Perspective

The patient was relieved that the diagnosis had been reached promptly, but was concerned about whether she would be able to return to work.

## 3. Discussion

In this case, a 45-year-old woman presented with anemia detected during health screening, and unintentional weight loss elicited during history-taking provided an important clue that led to the diagnosis of multiple myeloma. Although anemia was the only abnormality identified at health screening, actively recognizing weight loss as a red flag in the outpatient setting enabled prompt evaluation for malignancy rather than watchful waiting.

In premenopausal women, iron deficiency anemia often ranks high in the differential diagnosis because of chronic iron loss associated with menstruation. Indeed, anemia is not uncommon among Japanese women of reproductive age; one study reported a prevalence of anemia of 17.1% among women aged 20–49 years and suggested that menstrual bleeding and insufficient iron intake are major contributors to anemia in young women [3]. Iron deficiency is also the most common cause of anemia worldwide, and it is therefore reasonable that iron deficiency is considered first when anemia is encountered in premenopausal women [4]. However, in the present case, the MCV was within the normal range, and serum iron, total iron-binding capacity, and ferritin did not support iron deficiency. Vitamin B12 and folate deficiency were also absent. Therefore, even in women of reproductive age, anemia should not be attributed to menstruation alone; rather, red blood cell indices, iron metabolism, and vitamin status should be assessed, and causes other than iron deficiency should be considered when findings are atypical.

Unintentional weight loss is nonspecific but is an important red flag that may indicate serious disease, including malignancy. A systematic review in primary care reported that weight loss is associated with several cancer types and can serve as a trigger for cancer risk assessment, particularly when accompanied by other symptoms or laboratory abnormalities [5]. A more recent scoping review also described unexpected weight loss as a nonspecific symptom associated with undiagnosed cancer and examined how clinical guidelines address its diagnostic evaluation [6]. In the present case, the presence of unintentional weight loss as a red flag led us to prioritize an evaluation for malignancy, enabling timely diagnosis of multiple myeloma.

Current diagnostic criteria require clonal bone marrow plasma cells ≥ 10% or a biopsy-proven bony or extramedullary plasmacytoma, together with at least one myeloma-defining event (MDE), comprising CRAB features attributable to the plasma cell disorder or validated biomarkers of malignancy [7]. In the present patient, plasma cells accounted for 77.8% of marrow nucleated cells on aspiration, and the marrow was almost entirely replaced by plasma cells pathologically diagnosed as plasma cell myeloma, with clonality supported by the monoclonal protein findings. Regarding MDEs, the marrow plasma cell proportion exceeded 60%, and the involved/uninvolved serum free light-chain ratio was 4658.39 with an involved kappa level of 26,775.3 mg/L (≥100 mg/L), fulfilling two biomarker-defined MDEs. In addition, the anemia (hemoglobin 7.5 g/dL) was considered attributable to marrow replacement by plasma cells, because iron, vitamin B12, and folate deficiency and overt bleeding had been excluded and the erythropoietin level was not low. The osteolytic manubrial lesion was considered to represent myeloma bone disease; it was not biopsied because a histological diagnosis was not required once the bone marrow findings had established the diagnosis. Hypercalcemia and renal insufficiency meeting the CRAB thresholds were absent (calcium 9.2 mg/dL; creatinine 0.99 mg/dL). Therefore, the patient fulfilled the diagnostic criteria for multiple myeloma. However, the patient’s age was atypical. Multiple myeloma is a disease predominantly of older adults; SEER statistics report a median age at diagnosis of 69 years, and only 8.9% of newly diagnosed cases occur in patients aged 45–54 years [8]. Thus, in this 45-year-old woman, the disease could easily have been missed because anemia was the main abnormality and only mild nonspecific symptoms were present.

Health screening is widely used in Japan. The Comprehensive Survey of Living Conditions by the Ministry of Health, Labour and Welfare reported that 64.3% of adults aged 20 years or older had undergone a health checkup within the previous year [1]. However, health screening primarily detects laboratory or imaging abnormalities, whereas symptoms such as weight loss, fatigue, or pain may not be adequately assessed. Therefore, when patients present after an abnormal screening result, outpatient clinicians should not only recheck the abnormal value but also obtain a detailed history, including symptoms, their time course, and body weight changes. This case shows that even a common screening abnormality such as anemia can lead to the diagnosis of a serious disease, including multiple myeloma in a younger patient, when red flags are actively sought in outpatient practice.

## 4. Conclusions

This case demonstrates that multiple myeloma can present with anemia as the only abnormality detected at health screening in a younger woman, and that unintentional weight loss identified through detailed history-taking can serve as a critical diagnostic clue. When evaluating anemia detected during health screening, clinicians should go beyond simply rechecking laboratory values and instead systematically assess red flags, including weight loss, to avoid missing serious underlying conditions such as hematologic malignancies in younger patients.

## Data Availability

The data presented in this study are available from the corresponding author upon reasonable request. The data are not publicly available because they contain information that could compromise patient privacy.

## References

[1] Ministry of Health, Labour and Welfare of Japan Comprehensive Survey of Living Conditions: Status of Health Checkups and Ningen Dock Examinations. https://www.mhlw.go.jp/toukei/saikin/hw/k-tyosa/k-tyosa10/3-5.html.

[2] Kitayama R., Hioki A., Minami Y., Hayashi S., Kawai T. (2019). Characteristics and management of female examinees with low hemoglobin concentrations in Ningen Dock health checkups. Ningen Dock.

[3] Yamamoto K., Takita M., Kami M., Takemoto Y., Ohira T., Maeda M., Yasumura S., Sakai A., Hosoya M., Okazaki K. (2022). Changes in the proportion of anemia among young women after the Great East Japan Earthquake: The Fukushima Health Management Survey. Sci. Rep..

[4] Yamamoto K., Wang N., Takita M., Maeda Y., Tanimoto T., Crump A., Jiang Y., Zhao G. (2020). Iron deficiency anaemia: Its prevalence among women of reproductive age in Shanghai and Tokyo and links to body mass index. Cureus.

[5] Nicholson B.D., Hamilton W., O’Sullivan J., Aveyard P., Hobbs F.D.R. (2018). Weight loss as a predictor of cancer in primary care: A systematic review and meta-analysis. Br. J. Gen. Pract..

[6] Martinez-Gutierrez J., De Mendonca L., Ly P., Lee A., Hunter B., Manski-Nankervis J.A., Chima S., Daly D., Fishman G., Lim F.S. (2024). A scoping review of unexpected weight loss and cancer: Risk, guidelines and recommendations for follow-up in primary care. BJGP Open.

[7] Rajkumar S.V. (2024). Multiple myeloma: 2024 update on diagnosis, risk-stratification, and management. Am. J. Hematol..

[8] National Cancer Institute SEER Cancer Stat Facts: Myeloma. Surveillance, Epidemiology, and End Results Program. https://seer.cancer.gov/statfacts/html/mulmy.html.

